# Purine-rich low complexity regions are potential RNA binding hubs in the human genome

**DOI:** 10.12688/f1000research.13522.2

**Published:** 2019-05-09

**Authors:** Ivan Antonov, Yulia A. Medvedeva

**Affiliations:** 1 Institute of Bioengineering, Research Center of Biotechnology, Russian Academy of Sciences, Moscow, Russian Federation; 2 Department of Biological and Medical Physics, Moscow Institute of Physics and Technology, Dolgoprudny, Moscow Region, Russian Federation; 3 Vavilov institute of General Genetics, Russian Academy of Sciences, Moscow, Russian Federation

**Keywords:** MEG3 lncRNA, triple helix, triplex target sites (TTS)

## Abstract

Many long noncoding RNAs are bound to the chromatin and some of these interactions are mediated by triple helices. It is usually assumed that a transcript can form triplexes with a distinct set of genomic loci also known as triplex target sites (TTSs). Here we performed computational analyses of the TTSs that have been experimentally identified for particular RNAs. To assess the ability of these TTSs to bind other transcripts we developed a method to estimate the statistical significance of the predicted number of triplexes for a given RNA-DNA pair. We demonstrated that each DNA set included a subset of sequences that have a potential to form a statistically significant (adjusted *p*-value < 0.01) number of triplexes with the majority (>90%) of the analyzed transcripts. Due to the predicted ability of these DNA sequences to interact with a wide range of different RNAs, we called them "universal TTSs". While the universal TTSs were quite rare in the human genome (around 0.5%), they were  more frequent (>15%) among the MEG3 binding sites (ChOP-seq peaks) and especially among the shared Capture-seq peaks (40%). The universal TTSs were enriched with the purine-rich low complexity regions. Nowadays, the role of the chromatin bound RNAs in the formation of 3D chromatin structure is actively discussed. We speculated that such universal TTSs may contribute to establishing long-distance chromosomal contacts and may facilitate distal enhancer-promoter interactions. All the scripts and the data files related to this study are available at: https://github.com/vanya-antonov/universal_tts

## Introduction

Many human long noncoding RNAs are localized in the nucleus and can potentially participate in chromatin formation and remodeling ^1^. Recently, technologies such as ChIRP ^2^, ChRIP ^3^, ChOP ^4^, CHART ^5^, RAP ^6^, MARGI ^7^ and GRID ^8^ have been developed to map the genomic interacting sites of various lncRNAs. RNA can interact with chromatin by associating with DNA binding proteins, nascent transcripts, single-stranded or double-stranded DNA, forming R-loops or triple helices, respectively. Growing body of evidence shows that RNA-DNA triplex formation based on the Hoogsteen ^9^ base pairing rules plays important role in RNA-chromatin interactions. Several studies have provided *in vitro* and *in vivo* evidence for the existence and biological relevance of triplexes, including pRNA ^10^, Fendrr ^11^, Khps1 ^12^, PARTICLE ^13^, and MEG3 ^4^.

Computational analyses have revealed that a large population of triplex-forming motifs is present across the human genome with the majority of annotated genes containing at least one triplex target site (TTS), preferentially in regulatory gene regions ^14^, ^15^. Considering the large number of purine-rich sequences in the genome, triplex-mediated targeting of lncRNAs and associated proteins to distinct genomic loci is very likely a commonly used mechanism of gene regulation. Still, there are only a few bioinformatic studies of triplex-based RNA-DNA interactions on the genome-wide scale ^16^.

Here we analyze the genomic regions that are known to interact with the MEG3 lncRNA (the ChOP-seq peaks) or three different short oligos, corresponding to the DNA binding domains (DBDs) of MEG3 and GATA6-AS lncRNAs (the Capture-seq peaks). The current literature usually assumes that the triplex-based interactions have high sequence specificity and each triplex forming oligonucleotide (a transcript region) has a distinct set of genomic binding sites ("triplexome"). We investigate whether all the DNA sites capable of triplex formation are specific enough to be regulated by one particular RNA only or whether different transcripts may have shared TTSs. Our computational analysis revealed a group of genomic regions that may have a very high propensity for triplex formation with a wide range of different RNAs. Therefore, we named such DNA sequences "universal TTSs". We also attempted to reveal the features of these sequences that may be responsible for the observed phenomenon.

## Methods

The genomic coordinates of the 6837 MEG3 binding sites ^4^ (ChOP-seq peaks) were mapped from the hg19 to the hg38 human genome version using liftOver ^17^ (Nov 7, 2017 version). Next, from the 6800 successfully converted peaks we removed two cases corresponding to the genomic regions with ambiguous base-pairs (N) keeping the 6798 ChOP-seq peaks for the analysis. Additionally, to simulate the genomic background 6798 control regions with the lengths matching the selected ChOP-seq peaks were randomly sampled from the human genome using the bedtools ^18^ (version 2.27.1, see Supplementary Figure 1).

Triplex-based interactions were predicted by the Triplexator ^14^ (version 1.3.2) with the following parameters: -fr off -l 10 -e 10. These values were optimized so that the tool could predict binding between all three RNA-DNA sequence pairs that have been validated *in vitro* in the original study ^4^ ( Supplementary Table 1). To detect the statistically significant RNA-DNA interactions we developed a method to estimate a p-value from the number of predicted triple helices. Since the MEG3 peaks have different lengths, the expected number of triplexes (i.e. the parameter of the Poisson distribution) is computed based on the lengths of the input RNA and DNA sequences (see below).

The MEG3 binding sites have been identified in the triple negative breast cancer cell line BT-549 ^4^. To identify all the genes expressed in this cell line we used the RNA-seq data from the control knockdown experiment (ERR652847). The reads were aligned to the human genome (hg38) using HISAT2 ^19^ (version 2.1.0) and the number of reads corresponding to each GENCODE ^20^ (version 28) transcript was calculated by the HTSeq-count tool ^21^ (version 0.10.0). Next, the RPKM values were computed as RPKM = *C /*( *N × L*), where *C* is the number of reads aligned to all the transcript exons, *N* is the total number of mapped reads (in millions) and *L* is the transcript length (in kilobases). The most highly expressed (in terms of RPKM) isoform of each gene was considered only. Next, 153 expressed transcripts with the length and GC content similar to the MEG3 lncRNA (NR_002766.2) were selected using the RANN (version 2.6) R package ( Supplementary Figure 2). Additionally, 153 random RNA sequences were obtained by di-nucleotide shuffling the original MEG3 transcript using the uShuffle tool ^22^.

All the heatmaps were generated using the Complex-Heatmap ^23^ R package. The alignment of the RNA oligo sequences was obtained by the MUSCLE ^24^ (version 3.8). The locations of the RepeatMasker repeats in the human genome were downloaded from the UCSC Genome Browser ^17^.

### Calculation of the statistical significance of the predicted triplexes

For a given pair of RNA and DNA sequences, Triplexator outputs all the possible triple helices that satisfy the user-defined thresholds. Notably, the number of the predicted triplexes increases with the lengths of input sequences ( Supplementary Figure 3A). To account for this dependence the normalized number of triplexes (i.e. the "triplex potential" or *t _pot_*) is also computed by the Triplexator. Although this allows to compare triplexes predicted for RNA-DNA pairs with different lengths, it does not provide information about significance of these interactions.

To estimate the probability to observe a particular number of triplexes by chance (e.g. from the random sequences with the same lengths) we analyzed the average number of predicted triplexes between random sequences of various lengths. Namely, we considered four different RNA lengths ( *L _RNA_* = {500, 1000, 1500, 2000}) and ten different DNA lengths ( *L _DNA_* = {200, 400, ..., 1800, 2000}). For each of the 40 combinations of ( *L _RNA_*, *L _DNA_*), 100 random sequences with the length of *L _RNA_* and 100 random sequences with the length of *L _DNA_* were generated (with the equal frequencies for all the four nucleotides).

For each RNA-DNA pair triple helices were predicted by the Triplexator with the parameters optimized for MEG3 lncRNA ( -fr off -l 10 -e 10). Next, for every combination of ( *L _RNA_*, *L _DNA_*) the average number of predicted triplexes ( *λ*) was computed from all the 10000 predictions ( Supplementary Table 2). Finally, a linear regression model for *λ* was fitted to all the obtained values (adjusted *R* ^2^ = 87%, see Supplementary Figure 3B):

$$\lambda (L_{RNA},L_{DNA})=\theta_{0}+\theta_{1}\times L_{RNA}+\theta_{2}\times L_{DNA}\;(1)$$

where *θ*_0_ = −0.688, *θ*_1_ = 5.37 *×* 10 ^−4^ and *θ*_2_ = 6.03 *×* 10 ^−4^.

Thus, the statistical significance of the number of triple helices *N _tpx_*, predicted between RNA of length *L _RNA_* and DNA of length *L _DNA_*, can be estimated as follows. First, the expected average number of predicted triplexes ( *λ*) is computed from the equation (1). Next, the expected distribution of the number of predicted triplexes ( *H*_0_) is simulated by the Poisson distribution with the obtained value of *λ* ( Supplementary Figure 3C). Finally, the p-value of the observed number of triple helices ( *N _tpx_*) can be estimated as follows:

$$\text{P-value(}N_{tpx}\text{)}=P(X\geq N_{tpx}|X∼\text{Pois(}\lambda )\text{)}\;(2)$$

Importantly, the *N _tpx_* value is taken from the "Total (abs)" column of the triplex_search.summary file. The same value is used by the Triplexator to compute its "triplex potential" ( *t _pot_*). The ’Total (abs)’ is the total number of *all possible* triplexes that satisfy the user-defined thresholds (overlaps are allowed). Thus, for a single triplex longer than the minimal length (10 in our settings), the ’Total (abs)’ value may be greater than 1. For example, 11 bp DNA fragment 5’-GAGAGAGAGAG-3’ and 11 nt RNA oligo 5’-GAGAGAGAGAG-3’ can interact with each other forming one long anti-parallel triplex without mismatches. However, with the minimal allowed triplex length set to 10, the *N _tpx_* is equal to 3. This includes the long triplex of length 11 as well as the two triplexes of length 10 without the first or the last position of the long triplex. Therefore, a single long triplex is likely to produce a large *N _tpx_* value and, consequently, a statistically significant p-value.

### Calculation of purine and poly-purine contents

Due to the properties of the Watson-Crick base pairing model, the GC content of a sequence corresponding to the forward (+) DNA strand is equal to the GC content of the reverse (-) DNA strand. However, the GA content is more important for triplex based interactions because RNA can only form triple helices with the purines in the DNA. In contrast to the GC content, the GA content can be different between the DNA strands. Moreover, if one strand is purine rich, the other strand is automatically purine poor. For example, for the DNA sequence 5’-GGGGGAGA-3’ the purine content of the direct strand is 100%, while the other strand ( 3’-CCCCCTCT-5’) has no purines at all (i.e. its purine content is 0%).

Thus, we define the purine content of a given DNA fragment as the maximum value between the two strands, i.e.:

$$\text{GA-content}=\;{\;}_{s=\left\{+,-\right\}}^{\;\max}\frac{\text{NumPurines}({\text{DNA}}^{s})}{\text{Length}(\text{DNA})}\;(3)$$

where *DNA* ^+^ ( *DNA* ^-^) denotes the sequence corresponding to the forward (reverse) DNA strand and *NumPurines(DNA ^s^*) is the total number of G or A nucleotides present in the DNA strand *s*.

It should be noted that the purine content computed by the formula (3) is always *≥* 50%. To work with a measure that is defined from 0% to 100%, we introduce the *poly-purine content*. We define a poly-purine element *P ^s^* as a continuous stretch of 10 or more purines located on the DNA strand *s* (where *s* = {+, −}) . For a given DNA sequence that has *N* ^+^ poly-purine elements on the forward strand and *N* ^−^ poly-purine elements on the reverse strand, the poly-purine content is computed as follows:

$$\text{Poly-GA}\;\text{content}=\;{\;}_{s=\left\{+,-\right\}}^{\;\max}\frac{\sum_{i=1}^{N^{s}}\text{Length}(P_{i}^{s})}{\text{Length}(\text{DNA})}\;(4)$$

where *Length*( $P_{i}^{s}$) is the length of the poly-purine element *i* on the DNA strand *s*, i.e. according to the above definition Length( $P_{i}^{s}$) *≥* 10.

## Results

**Figure 1.**
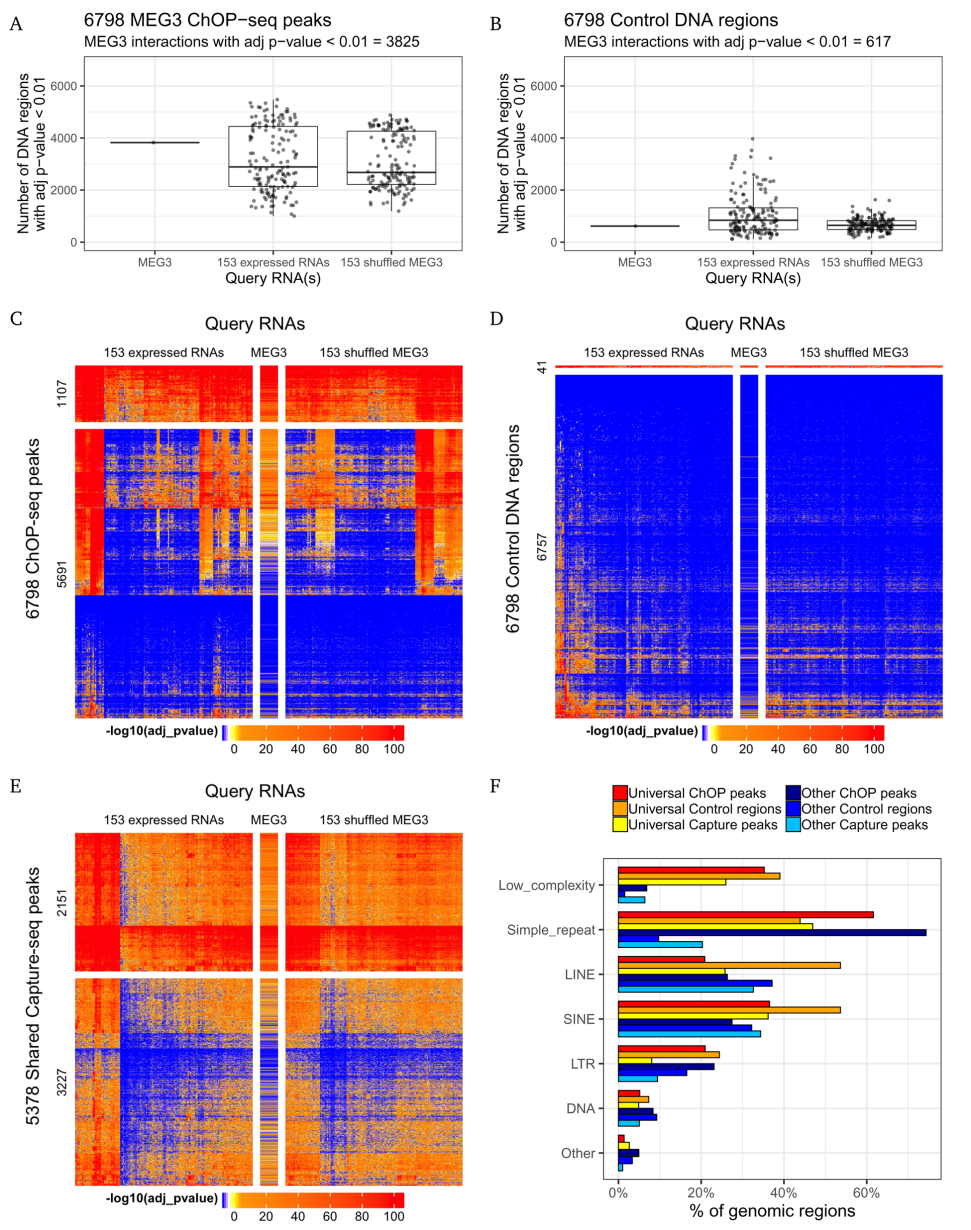
( **A**, **B**) The number of the DNA sequences from ( **A**) the ChOP-seq or ( **B**) the control genomi set with the statistically significant number of predicted triplexes for different query RNAs (the black dots). ( **C**, **D**, **E**) The heat maps of the *–* log _10_ (adjusted p-value) corresponding to the predicted triplexes between the 307 different query RNAs (columns) and ( **C**) all the ChOP-seq peaks, ( **D**) the control genomic sites or ( **E**) the Shared Capture-seq peaks (rows). The universal TTSs were identified based on their interactions with the 153 expressed transcripts (left part of each heat map) and visualized as a separate (top) cluster. The MEG3 column was intentionally drawn wider. The blue color corresponds to the RNA-DNA pairs with adjusted p-value = 1 (including cases where no triplexes were predicted). ( **F**) Repeat classes present in different sets of genomic regions.

We used Triplexator to predict possible triple helices between the full length MEG3 transcript and the 6798 experimentally identified ChOP-seq peaks as well as 6798 control DNA regions (see Methods). The genomic sites with a statistically significant number of triplexes were identified in each DNA set by our custom probabilistic approach (see Methods). As anticipated, more statistically significant (Bonferroni adjusted p-value < 0.01) interactions with the MEG3 lncRNA were predicted for the ChOP-seq peaks than for the control regions. Namely, the interactions with the 3825 (56.3%) ChOP-seq peaks were classified as statistically significant ( Figure 1A, left) while there were only 617 (9.1%, odds ratio test p-value < 2.2 *×* 10 ^−16^) such cases among the control DNA regions ( Figure 1B, left). Since the ChOP-seq method has detected RNA contacts with the chromatin (and not the naked DNA) the obtained binding sites can correspond to several different interaction mechanisms including direct RNA-DNA interactions via triple helices or R-loops, RNA-RNA hybridization with nascent transcripts as well as bindings to nuclear proteins. This may be the reason that many MEG3 binding sites did not produce statistically significant predictions with the MEG3 lncRNA. Therefore, these results supported the original conclusion that the MEG3 lncRNA is able to directly interact with the genomic DNA via triple helices.

To check the ability of other RNAs to form triplexes with MEG3 binding sites, we applied Triplexator to a set of 153 expressed transcripts (see Methods). Surprisingly, 65 analyzed RNAs showed results similar to MEG3 lncRNA – they were predicted to form statistically significant interactions with the majority (> 50%) of the ChOP-seq peaks ( Figure 1A, middle). To further investigate possible interactions with the ChOP-seq peaks, 153 artificial sequences were generated by di-nucleotide shuffling of the MEG3 transcript (see Methods). Strikingly, these random "RNAs" produced statistically significant number of triplexes with 39% and 9% of the ChOP-seq peaks and control DNA regions, respectively ( Figure 1A, B, right). These results indicated that the set of the ChOP-seq peaks was different from the randomly sampled genomic sites in that it contained a number of DNA sequences that may be able to interact not only with the MEG3 lncRNA, but with other RNAs as well.

Based on these observations we hypothesized that some of the MEG3-bound genomic sites may be ’universal’, i.e. they may have a potential to form multiple triplexes with a number of different RNAs. Analysis of the Triplexator predictions obtained for the 153 expressed RNAs revealed 1107 (16.3%) ChOP-seq peaks that were predicted to form statistically significant number of triplexes with more than 90% of the analyzed transcripts ( Supplementary Figure 4A). In contrast, the genomic background contained only 41 (0.6%) such sites ( Supplementary Figure 4B). Due to the predicted ability of these genomic regions to form triple helices with various RNAs, we called them "universal triplex target sites (TTSs)". Notably, the identified universal TTSs produced strong p-values for the MEG3 lncRNA as well as for the 153 MEG3 shuffled sequences ( Figure 1C, D). Thus, according to our predictions some of the DNA sequences were more prone to formation of triple helices with different long RNAs and a number of such genomic regions were present among the experimentally identified MEG3 binding sites (ChOP-seq peaks).

To further investigate the predicted ability of the universal TTSs to bind various RNAs we analyzed the results of the recent Capture-seq experiment ^25^. This *in vitro* study has determined genomic binding sites of three different short RNA oligos that corresponded to the DNA-binding domains (DBDs) of the MEG3 and GATA6-AS lncRNAs. Namely, the *MEG3_13_41*, *GATA6_AS_78_118* and *MEG3_839_890* oligos were 28, 40 and 48 nt long, respectively. Since the experiment has been performed on the RNA- and protein-free ("naked") genomic DNA, the majority of the identified interactions have been assumed to be direct and mediated by triple helices. Comparison of the genomic coordinates corresponding to the identified target DNA fragments demonstrated that most of the interactions were specific to one oligo only ( Supplementary Figure 5). This can be explained by the fact that the oligo sequences had limited similarity with each other (the identities between the *MEG3_13_41*- *GATA6_AS_78_118*, *MEG3_839_890*- *GATA6_AS_78_118* and *MEG3_13_41*- *MEG3_839_890* oligo pairs were 33%, 40% and 25%, respectively – Supplementary Figure 6). Still, 5379 genomic regions were captured by each of the three oligos ( Supplementary Figure 5). Thus, we expected that this set of ’shared Capture-seq peaks’ can be enriched with the potential universal TTSs. To check this we predicted their possible interactions with the 153 RNA sequences that were used in the analysis of the ChOP-seq peaks (see above). Indeed, 2151 (40%) shared Capture-seq peaks were classified as universal TTSs – they had statistically significant number of triplexes with most (> 90%) of the analyzed transcripts ( Figure 1E and Supplementary Figure 4C). Therefore, the fact that the experimentally identified set of shared Capture-seq peaks contained such a high fraction of the universal TTSs indirectly confirmed the predicted property of these special genomic loci.

Finally, we attempted to reveal the features of the universal TTSs that may allow them to interact with several different RNAs. For this purpose we compared sequence composition of the universal and all the other (i.e. non-universal) genomic regions from each set. While the GC content of the universal and non-universal DNA sequences were similar, the universal TTSs had higher purine (G or A) and, especially, poly-purine content (see Supplementary Figure 7 and Methods for the definitions). To find out the origin of these poly-purine elements we analyzed the classes of the overlapping genomic repeats. All three sets of the universal TTSs were enriched with the purine-rich low complexity regions, LCRs ( Figure 1F and Supplementary Figure 8). Additional analysis of several universal TTSs confirmed that these LCRs were predicted to form multiple triple helices with the majority of the analyzed transcripts (see Supplementary Figure 9 for representative cases). Therefore, the presence of the purine-rich low complexity elements was the characteristic property of the universal TTSs that potentially allowed them to interact with a wide range of different RNAs. All together our results suggested the existence of a special type of genomic loci that may function as RNA-binding hubs.

## Discussion

The importance of triplex-dependent gene regulation in the genomes of higher organisms is becoming a generally accepted concept. Here we performed a large-scale bioinformatic analysis of the genomic regions (ChOP-seq and Capture-seq peaks) that have been shown experimentally to interact with particular RNAs (MEG3 lncRNA or short oligos). To filter out not significant Triplexator predictions, the statistical significance of every RNA-DNA interaction was estimated from the Poisson distribution. To our surprise for some genomic regions (that we called "universal triplex target sites") Triplexator predicted statistically significant (adjusted p-value < 0.01) number of triplexes with the majority (> 90%) of the analyzed transcripts. According to our analysis universal TTSs are quite rare in the human genome – there were only 0.6% of them among the 6798 randomly sampled regions. On the other hand, 16.3% of the experimentally identified MEG3 binding sites (ChOP-seq peaks) were classified as universal TTSs. Additionally, genomic regions that have been shown to form triplexes with three different oligos (shared Capture-seq peaks) contained 40% of the universal TTSs. All three sets of the identified universal TTSs were enriched with the purine rich low complexity regions.

The theoretical possibility of the universal TTS existence comes from the degeneracy of the Hoogsteen rules ^9^. In fact, the triplex-based interaction can be formed in both orientations (parallel and anti-parallel) and it involves only purines (G or A) in the DNA. Additionally, the DNA guanine and adenine can bind to RNA guanine and uracil, respectively, in both orientations while the A::A pairing occurs in the anti-parallel orientation only. This makes the long poly-purine elements a possible targets for a number of different RNA oligos.

One of the possible and actively discussed roles of the chromatin bound RNAs (including lncRNAs) is to bring different chromosomal parts together to enable the remote DNA-DNA contacts ^8^. Moreover, it has recently been shown that RNAs originating from super-enhancers form triplexes at distant regions ^26^. Therefore, it is possible that universal TTSs may facilitate distal enhancer-promoter interactions via engagement with the same enhancer RNA. In line with this hypothesis, we observed the statistical significant enrichment of the universal Capture-seq peaks near (< 1 kb) the transcription start sites (TSSs) of the annotated genes ( Supplementary Figure 10A). However, the computationally predicted universal ChOP-seq and background TTSs did not have such trend ( Supplementary Figure 10B,C). Thus, the experimentally identified shared Capture-seq peaks may be more suitable for subsequent functional validation of the universal TTSs in living cells.

Importantly, the current computational analysis has a number of limitations. Namely, the triplex-based interactions of the full length transcripts were predicted without taking their secondary structure into account. We are not aware of any bioinformatics tools that would be able to produce such predictions. Moreover, cellular localization of the 153 selected expressed transcripts as well as DNA binding proteins and chromatin compaction were not considered. Therefore, our simulations are more similar to the *in vitro* Capture-seq experiments with short oligos than to the interactions of long transcripts with the chromatin inside the nucleus. Comprehensive identification of all the RNA-DNA interactions obtained by high throughput experimental methods may clarify the predicted functionality of the universal TTSs in the cell. Although a few methods for this task have recently been developed, the length of the sequencing reads (e.g. about 40 bp of DNA in case of GRID-seq) does not allow to reliably determine interactions with the long low complexity regions (including universal TTSs). We are looking forward to the new high quality experimental data to gain further insight into the triplex-based RNA-chromatin interactions *in vivo*.

## Data availability

### Underlying data

Zenodo: vanya-antonov/universal_tts: The initial release of the code, data files and images related to universal TTSs. http://doi.org/10.5281/zenodo.2654800 ^27^

This project contains the following underlying data:

- universal_tts-v1.0.0.zip?download=1.zip
  – data (folder containing underlying data, description of individual files can be found in Supplementary Table 3)

### Extended data

Zenodo: vanya-antonov/universal_tts: The initial release of the code, data files and images related to universal TTSs. http://doi.org/10.5281/zenodo.2654800 ^27^

This project contains the following extended data:

- universal_tts-v1.0.0.zip?download=1.zip
  – images_R (folder containing R scripts to generate figures)
  – scripts (folder containing scripts to compute p-values based on the Triplexator predictions)

Data and code are available under the terms of GNU General Public License version 3 (GPL-3.0).

## Supplementary material

Supplementary File 1. File containing Supplementary figure 1–Supplementary figure 10, and Supplementary table 1–Supplementary table 3.
