# Supplementary material for: Purine-rich low complexity regions are potential RNA binding hubs in the human genome
Source: F1000Res. 2019 May 9;7:76. Originally published 2018 Jan 17. [Version 2] doi: 10.12688/f1000research.13522.2 (PMC6518440; doi:10.12688/f1000research.13522.2)
Supplement: Supplementary file 1 [file f1000research-7-21026-s0000.tgz › a0b6e7f5-2cc9-4a47-86a2-e2e5ebb21945_Supplementary_File_1.pdf]

1 Supplementary figures

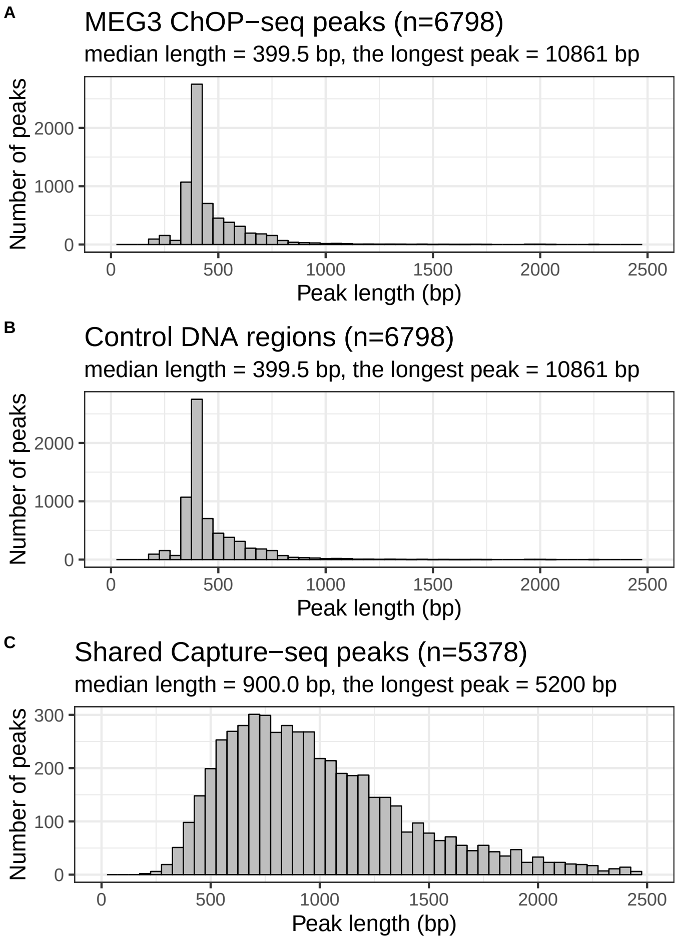

Supplementary Figure 1: The distributions of lengths of (A) the ChOP-seq peaks, (B) the selected control DNA regions and (C) the shared Capture-seq peaks.

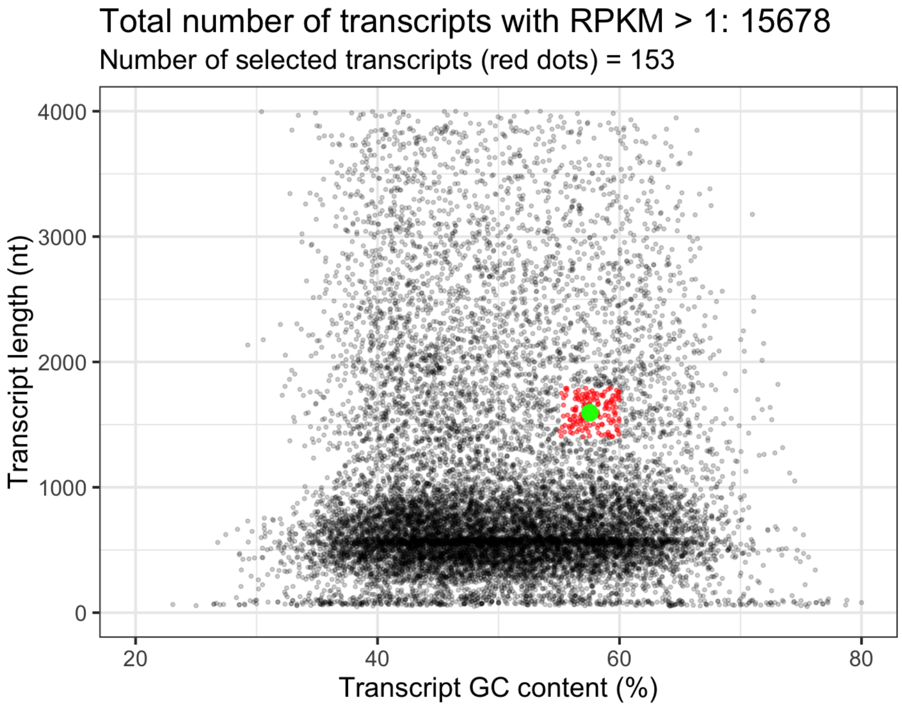

Supplementary Figure 2: Selecting expressed transcripts similar to the MEG3 by the length and GC content. The MEG3 isoform NR_002766.2 (indicated by the large green dot on the plot) has the length = 1595 nt and GC-content = 57.55%. Each gene is represented by its most highly expressed isoform (black dots). Among all the transcripts with RPKM > 1, 153 RNAs with the length between 1400 nt and 1800 nt and GC content between 55% and 60% were selected (red dots).

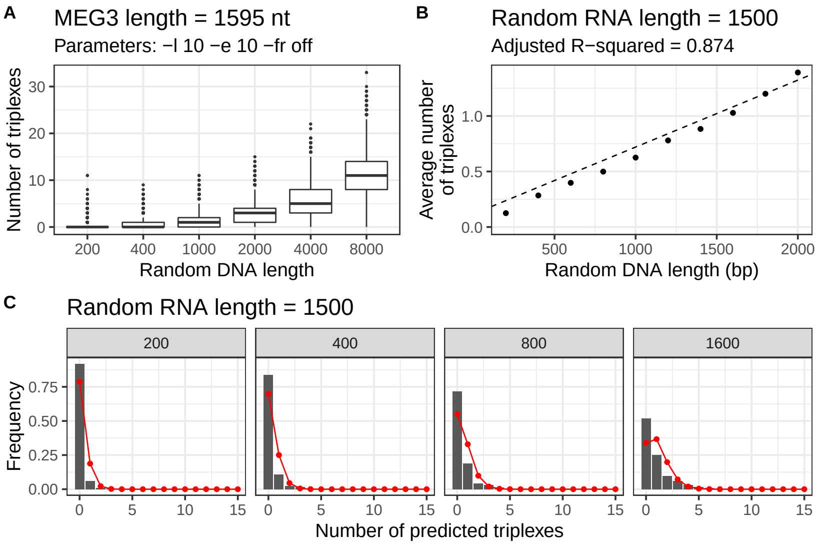

Supplementary Figure 3: (a) Distributions of the number of predicted triplexes between the MEG3 lncRNA (NR_002766.2) and random DNA sequences of different lengths. (b) Dependence of the average number of predicted triplexes on the length of the random DNA sequences (the random RNA length is fixed). The dashed line corresponds to the fitted linear model. (c) Distributions of the number of triplexes predicted between random RNA and random DNA sequences. The DNA lengths are indicated in gray boxes above each histogram while the random RNA length remains the same in all the graphs. The red line in each plot corresponds to the Poisson distribution with the lambda parameter predicted from the linear regression model (see Methods). Each distribution was built based on the Triplexator predictions for 10,000 random RNA-DNA pairs.

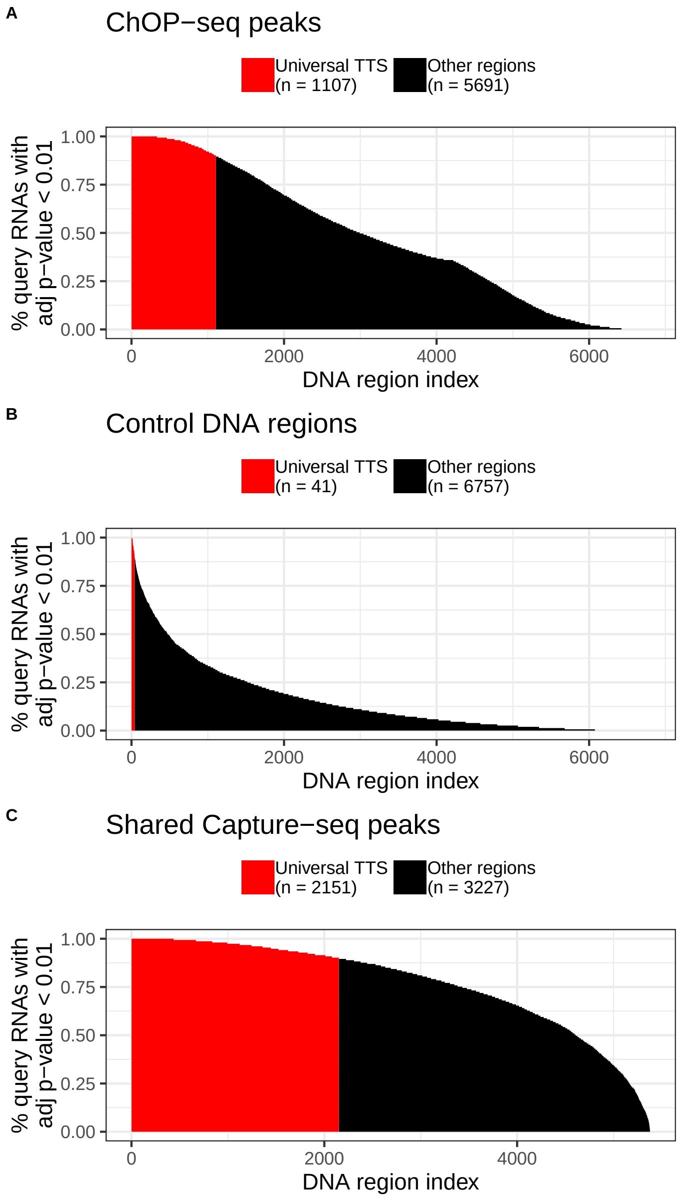

Supplementary Figure 4: Identification of the universal TTSs among (A) ChOP-seq peaks, (B) control DNA regions or (C) shared Capture-seq peaks. Y-axis: the percentage of the 153 selected transcripts predicted to form a statistically significant number of triplexes for each DNA region. X-axis: the DNA regions sorted by this percentage. The bars corresponding to the genomic sites classified as universal TTSs are colored by red.

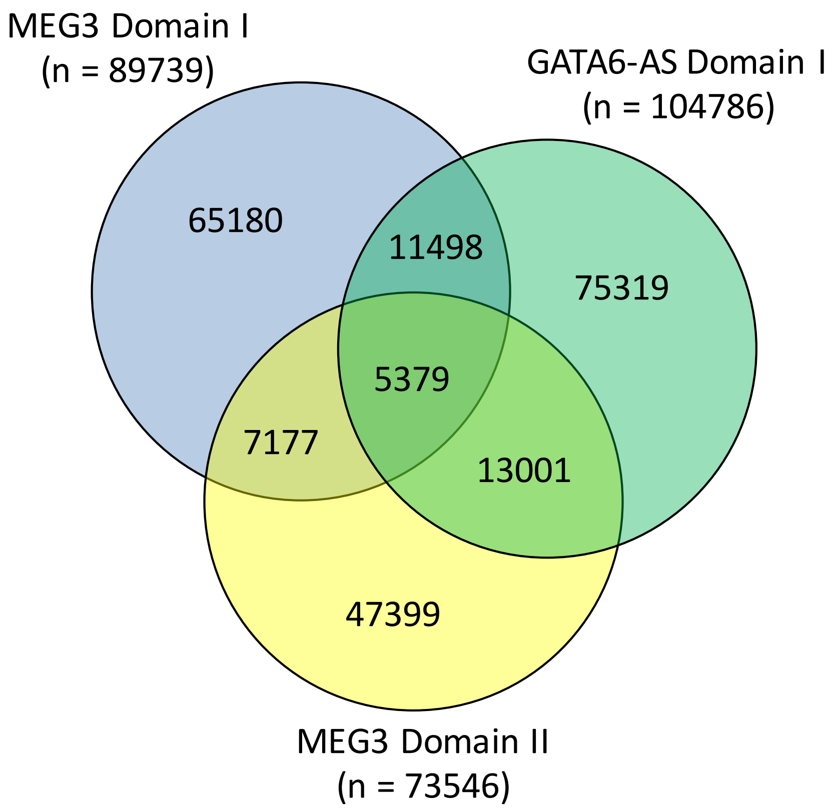

Supplementary Figure 5: Overlap between peaks identified in independent Capture-seq experiments performed for three different RNA oligos. It should be noted that the sum of the numbers inside each circle may not be exactly equal to the total number of peaks identified in the experiment because a peak corresponding to one oligo can overlap with several different peaks corresponding to another oligo.

```
CLUSTAL multiple sequence alignment by MUSCLE (3.8)


MEG3_839_890         CAGUCCCUUCCCACCCCUCUUGCUUGUCUACUGUCUAUUUAUUCUCCA
MEG3_13_41           GAC------------------GGCGGAGAGCAGAGAGGGAGCGCGC--
GATA6_AS_78_118      UGCUCUGCGCCCCCCC-----GCCCCCCAACCCCCGCCUAGCCCC---
                                          *        *            *

#  Percent Identity  Matrix
#
#

     1: MEG3_839_890     100.00   25.00   40.00
     2: MEG3_13_41        25.00  100.00   33.33
     3: GATA6_AS_78_118   40.00   33.33  100.00
```

Supplementary Figure 6: The alignment and the pairwise identity matrix of the three RNA oligos used in the Capture-seq experiments.

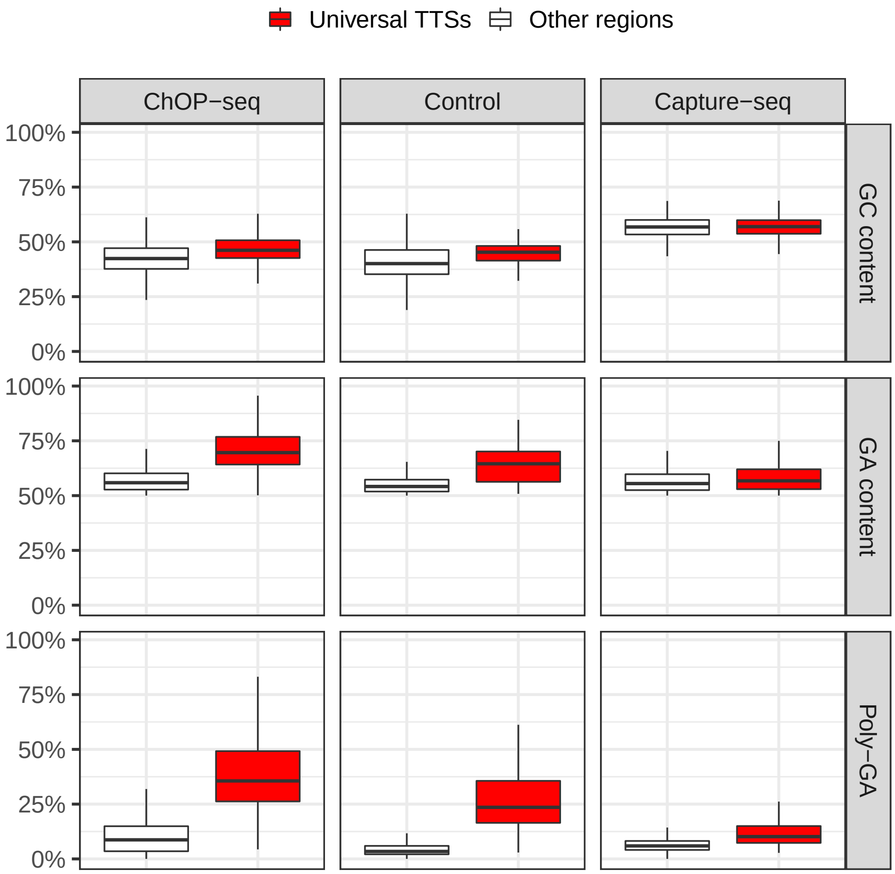

Supplementary Figure 7: Comparison of the sequence features (rows) of the universal TTSs (red) and the other genomic regions (white) identified in the three DNA sets (columns). See Methods for the equations to compute the GA-content and Poly-GA-content.

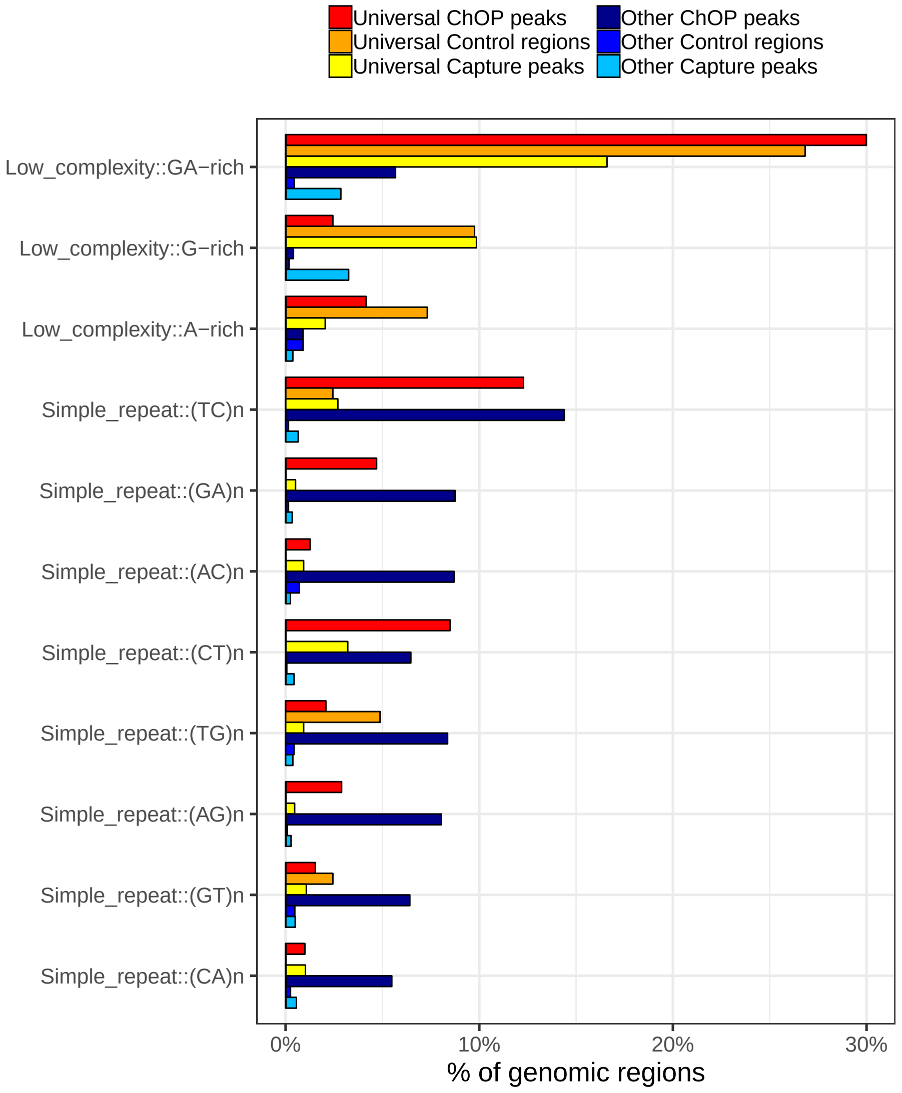

Supplementary Figure 8: Low complexity and simple repeat subtypes present in different sets of genomic regions. Subtypes found in $\geq 5\%$ of the DNA sequences from at least one set were included only.

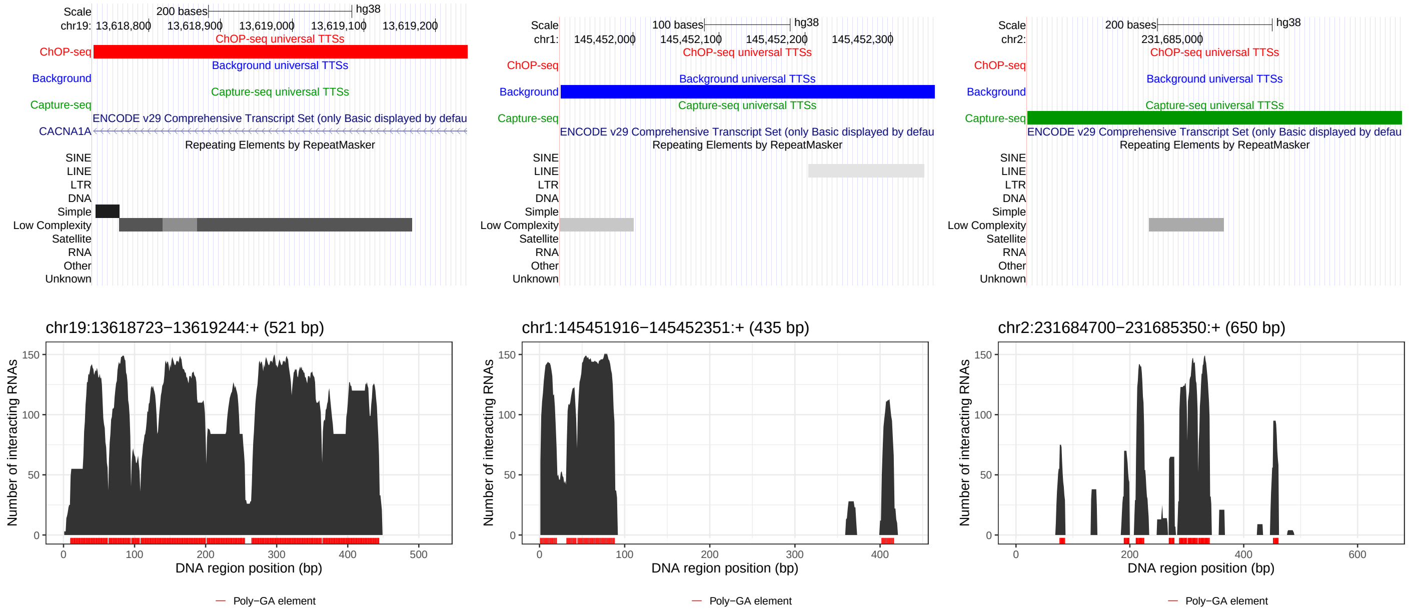

(a) Universal ChOP-seq peak (b) Universal Background region (c) Universal Capture-seq peak

Supplementary Figure 9: Coverage of the three DNA regions by the triple helices. The black bars indicate the number of the 153 selected expressed RNAs with at least one triplex predicted at each DNA position. The red lines below each plot indicate the locations of the poly-purine elements ($\{G|A\}_n, n \geq 10$). The UCSC Genome Browser screen shots above the plots show the overlapping RepeatMasker repeats for the corresponding genomic loci.

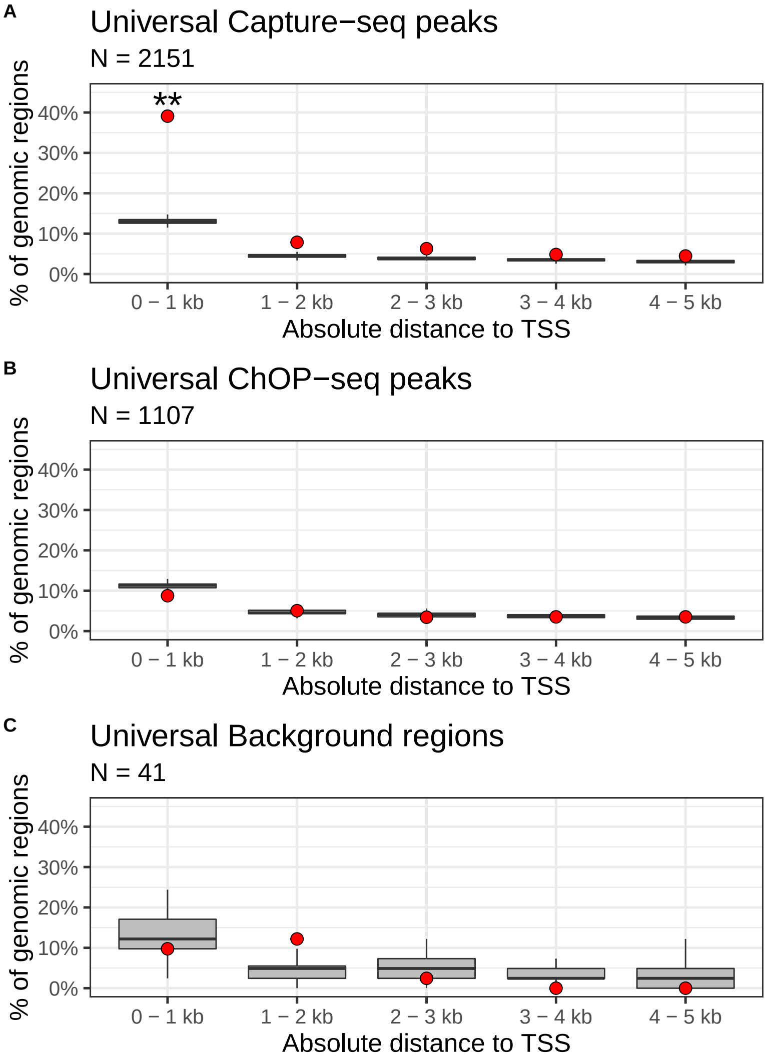

Supplementary Figure 10: Locations of the three sets of the universal TTSs relative to the transcription start sites (TSSs) of the annotated GENCODE transcripts. The red dots indicate the percentage of the universal TTSs that are located within a particular distance interval from the nearest TSS. The boxplots are the distributions of the expected percentages that were obtained by randomly sampling 100 sets of genomic regions with lengths matching the corresponding universal TTSs. **empirical p-value < 0.01.

2 Supplementary tables

Supplementary Table 1: Optimization of the Triplexator parameters using the *in vitro* validated interactions between the MEG3 DNA binding domain (MEG3_19_38) and the three DNA fragments. Parameter description: `-fr off` – do not filter low complexity and repeat regions in the sequence data, `-l 10` – specifies the minimum length of triplex (TTS-TFO pair), `-e 10` – set the maximal error-rate in % tolerated (default 5). The input sequences were: CGGA-GAGCAGAGAGGGAGCG (MEG3_19_38), AGAGAGAGGGAGAGAG (TGFBR1_peak), CAGAGAGCAGAGAGAGAGA (TGFB2_peak), AGAGAGGGAGAG (SMAD2_peak).

**Triplexator parameters**	**MEG3_19_38 vs TGFBR1_peak**	**MEG3_19_38 vs TGFB2_peak**	**MEG3_19_38 vs SMAD2_peak**
-fr off -l 12 -e 5	No prediction	No prediction	No prediction
-fr off -l 10 -e 5	TFO: 3'- GAGGGAGAGA -5'<br>|||||||||| TTS: 5'- GAGGGAGAGA -3'<br>3'- CTCCCTCTCT -5'	No prediction	No prediction
-fr off -l 10 -e 10	TFO: 3'- GcGAGGGAGAGA -5'<br>|*|||||||||| TTS: 5'- GAGAGGGAGAGA -3'<br>3'- CTCTCCCTCTCT -5'	TFO: 3'- GAGGGAGAGA -5'<br>|||*|||||| TTS: 5'- GAGAGAGAGA -3'<br>3'- CTCTCTCTCT -5'	TFO: 3'- GcGAGGGAGAG -5'<br>|*||||||||| TTS: 5'- GAGAGGGAGAG -3'<br>3'- CTCTCCCTCTC -5'

Supplementary Table 2: The observed average number of triplexes (λ) predicted between random RNA and DNA sequences of different lengths.

		RNA length (nt)			
		500	1000	1500	2000
DNA length (bp)	200	0.04	0.09	0.12	0.18
	400	0.11	0.21	0.28	0.43
	600	0.13	0.31	0.40	0.61
	800	0.17	0.38	0.50	0.72
	1000	0.20	0.46	0.63	0.93
	1200	0.25	0.60	0.78	1.17
	1400	0.31	0.68	0.88	1.38
	1600	0.35	0.76	1.03	1.52
	1800	0.39	0.90	1.20	1.77
	2000	0.46	1.04	1.39	2.07

Supplementary Table 3: Information about some of the supplementary data files.

File(s)	Related Figures/Tables	Description
`chop_seq_hg19.bed`<br>`chop_seq_hg38.bed`<br>`background_hg38.bed`<br>`capture_seq_hg38.bed`	Figure 1<br>Supplementary Figure 1	Coordinates of the original (hg19-based) and the converted (hg38-based) MEG3 ChOP-seq peaks, selected control DNA regions and shared Capture-seq peaks.
`meg3.fasta`<br>`expressed_trxs.fasta`<br>`meg3_shuffled.fasta`	Figure 1	The sequences of all the 307 queries – the MEG3 lncRNA, 153 expressed transcripts and 153 random sequences obtained by MEG3 di-nucleotide shuffling.
`pvalue_chop_seq.txt.gz`<br>`pvalue_background.txt.gz`<br>`pvalue_capture_seq.txt.gz`	Figure 1<br>Supplementary Figure 4	Matrices of the raw p-values between 307 query RNAs and different sets of DNA sequences.
`uni_tts_chop_seq_hg38[.bed|.fasta]`<br>`uni_tts_background_hg38[.bed|.fasta]`<br>`uni_tts_capture_seq_hg38[.bed|.fasta]`	Figure 1<br>Supplementary Figures 4, 10	The coordinates and sequences of the universal TTSs predicted in three different sets of genomic regions.
`random_simulations.txt.gz`	Supplementary Figure 3<br>Supplementary Table 2	Triplexator predictions for random sequences of different lengths.
`all_expressed_trxs.txt`	Supplementary Figure 2	Properties (length and GC content) and the RPKM values of the transcripts expressed in BT-549 cells.
`chop_seq[.len_gc|.len_purine]`<br>`background[.len_gc|.len_purine]`<br>`capture_seq[.len_gc|.len_purine]`	Supplementary Figure 7	Properties (length, GC, purine and poly-purine content) of the DNA sequences.
