# Supplementary material for: Purine-rich low complexity regions are potential RNA binding hubs in the human genome
Source: F1000Res. 2019 May 9;7:76. Originally published 2018 Jan 17. [Version 2] doi: 10.12688/f1000research.13522.2 (PMC6518440; doi:10.12688/f1000research.13522.2)
Supplement: Peer review history

# Peer review: Reviewer response for version 2

**DOI:** 10.5256/f1000research.21026.r48244

**Published:** 2019-05-21

**Authors:** Hao Zhu

**Affiliations:** 1 Bioinformatics Section, Southern Medical University, Guangzhou, China

The manuscript is improved and well-written, and the idea of "universal TTSs" is interesting, but there are still several weak points. These points make some of my concerns remain but are not as serious as before. So, I think it is better to let readers make their own judgement.

 First, the authors analyzed basically only the data of MEG3, which makes the basis of the conclusion weak. The authors say, "To check the ability of other RNAs to form triplexes with MEG3 binding sites, we applied Triplexator to a set of 153 expressed transcripts". The specific 153 ones (given that there are ~20,000 annotated lncRNA genes) make the following "65 analyzed RNAs show results similar to MEG3 lncRNA" quite specific.

 Second, both the ChOP-seq data and Triplexator results may have defects.  For example, the authors say, "the number of the predicted triplexes increases with the lengths of input sequences". Why? Theoretically, if the longer sequences do not contain more TFO, the number of the triplexes should not increase.

I have read this submission. I believe that I have an appropriate level of expertise to confirm that it is of an acceptable scientific standard.

# Peer review: Reviewer response for version 2

**DOI:** 10.5256/f1000research.21026.r48245

**Published:** 2019-05-13

**Authors:** Ingrid Grummt

**Affiliations:** 1 Division of Molecular Biology of the Cell II , DKFZ-ZMBH-Allianz, German Cancer Research Center (DKFZ), Heidelberg, Germany

The authors have responded to most of my concerns. They increased the number of RNA sequences and included published data in their analysis. Though some concerns are left, the overall conclusion of this study – that is, purine-rich sequences interact with various different RNAs – supports the notion that regulatory RNAs may target transcriptional co-activators to distinct genomic loci via Hoogsteen interactions with purine-rich gene sequences.

I have read this submission. I believe that I have an appropriate level of expertise to confirm that it is of an acceptable scientific standard.

# Peer review: Reviewer response for version 1

**DOI:** 10.5256/f1000research.14683.r29953

**Published:** 2018-02-05

**Authors:** Hao Zhu

**Affiliations:** 1 Bioinformatics Section, Southern Medical University, Guangzhou, China

Many lncRNAs can bind to DNA sequences by forming triplexes (the binding sites are often called TTS, triplex-targeting sites). Whether there are “universal TTS” as described here is interesting and unreported, and deserves a careful investigation. But I have a major concern about the work: the authors reach the conclusion upon too few examples. Also, why these lncRNAs (BE2L6, LILRA3, HMOX1) were chosen (randomly or selected for some reasons)?

 A few others issues should also be addressed. First, what is the relationship between the “universal TTSs” and base-pairing rules is untouched. For example, do the universal TTSs allow many lncRNAs to bind to them using the same rules? If very different rules are involved, what does this mean? To some extent, binding upon different rules indicates lncRNA specific TTSs, instead of universal TTSs. Second, I feel that the genomic regions used to sum scores are unreasonably long (3000 bp). Finally, it is said that “the median Triplexator SumScores were 48 and 25, respectively (p-value=5.2e-100)”. Statistically, the difference is significance, but biologically might not. I think 48 is not that large and 25 is not that small.

I have read this submission. I believe that I have an appropriate level of expertise to state that I do not consider it to be of an acceptable scientific standard, for reasons outlined above.

# Response

**Published:** 2019-05-10

**Authors:** Ivan Antonov

**Affiliations:** Research Center of Biotechnology, RAS, Russian Federation

Many lncRNAs can bind to DNA sequences by forming triplexes (the binding sites are often called TTS, triplex-targeting sites). Whether there are “universal TTS” as described here is interesting and unreported, and deserves a careful investigation. But I have a major concern about the work: the authors reach the conclusion upon too few examples. Also, why these lncRNAs (BE2L6, LILRA3, HMOX1) were chosen (randomly or selected for some reasons)?

**In the current version we have significantly increased the number of query RNAs (i.e. 153 expressed transcripts and 153 random sequences obtained by di-nucleotide shuffling of MEG3 lncRNA). The 153 transcripts that we use now were chosen so that their lengths and GC contents were similar to the MEG3 lncRNA.**

 1) A few others issues should also be addressed. First, what is the relationship between the “universal TTSs” and base-pairing rules is untouched. For example, do the universal TTSs allow many lncRNAs to bind to them using the same rules? If very different rules are involved, what does this mean? To some extent, binding upon different rules indicates lncRNA specific TTSs, instead of universal TTSs.

**Our preliminary analysis indicated that different RNAs interacted with universal TTSs via mixed (G or U) motif a little bit more frequently than via the purine or pyrimidine motifs. Importantly, all the analyzed transcripts were predicted to form a lot of triple helices (using different RNA motifs) with the universal TTSs. This is what makes uTTS special rather than specific motifs .**

 2) Second, I feel that the genomic regions used to sum scores are unreasonably long (3000 bp).

**In the current version we decreased the genomic region lengths by considering the exact ChOP-seq and Capture-seq peaks.**

 3) Finally, it is said that “the median Triplexator SumScores were 48 and 25, respectively (p-value=5.2e-100)”. Statistically, the difference is significance, but biologically might not. I think 48 is not that large and 25 is not that small.

**We now use p-values instead of SumScore to estimate the statistical significance of each RNA-DNA interaction.**

# Peer review: Reviewer response for version 1

**DOI:** 10.5256/f1000research.14683.r29955

**Published:** 2018-01-26

**Authors:** Andrey A. Mironov

**Affiliations:** 1 Department of Bioengineering and Bioinformatics, Moscow State University, Moscow, Russian Federation; 2 Institute for Information Transmission Problems, RAS (Russian Academy of Sciences), Moscow, Russian Federation

The manuscript describes an application of the Triplexator software for search possible binding sites of the imprinting related MEG3 linc RNA on the human genome. The authors give a good example of the statistical analysis of the results. The main paper about this software has 62 references (google scholar data). Most of them have only reference to the software and only a few of them used the Triplexator. Only a few reports show a success story about the application of the Triplexator software and comparison the results with an experiment. In some papers, a significant enrichment of triplex targets on regions of interest was found. But they did not analyze the specificity of the predicted triplex formation. The current paper focused on a specificity of the Triplexator predictions. The authors got unexpected results that the Triplexator gives many non-specific hits for the case.

 Comments:

1. Description of similar transcripts and the parameters of the Triplexator software should be rearranged because the appearance of some RNA names before definition sounds strange. The parameters of the Triplexator software contains a reference to the BE2L6 RNA while the description of the control RNA set has a reference on UBE2L6.
2. The di-nucleotide shuffling seems more adequate for RNA analysis.
3. In one paper ^1^ the Triplexator software also was used for analysis of MEG3 RNA-DNA contacts. The comparison of the obtained results with the results of given manuscript should be provided. Seems in current manuscript a more accurate analysis with good controls was provided.
4. It would be good to look at the practice of using the program on literature and make sure that the program has a sufficiently low specificity.

I have read this submission. I believe that I have an appropriate level of expertise to confirm that it is of an acceptable scientific standard.

## References

[1] MondalTSubhashSVaidREnrothSUdaySReiniusBMitraSMohammedAJamesARHobergEMoustakasAGyllenstenUJonesSJGustafssonCMSimsAHWesterlundFGorabEKanduriC: MEG3 long noncoding RNA regulates the TGF-β pathway genes through formation of RNA-DNA triplex structures. *Nat Commun*.2015;6: 10.1038/ncomms8743 7743 10.1038/ncomms8743 26205790PMC4525211

# Response

**Published:** 2019-05-10

**Authors:** Ivan Antonov

**Affiliations:** Research Center of Biotechnology, RAS, Russian Federation

**Dear reviewer,**

****

**We would like to apologise for a significant delay with the reply to all the comments. To implement the changes suggested by the reviewers we had to completely redesign our study and significantly increase the number of analyzed RNAs. Particularly, we developed a new method to estimate the statistical significance of the number of triplexes predicted for each RNA-DNA pair. Moreover, we analyzed the results of the recently published Capture-seq experiment that identified interactions of three different RNA oligos with "naked" DNA. We hope that all these analyses improved our study.**

 1) Description of similar transcripts and the parameters of the Triplexator software should be rearranged because the appearance of some RNA names before definition sounds strange. The parameters of the Triplexator software contains a reference to the BE2L6 RNA while the description of the control RNA set has a reference on UBE2L6.

**We have made the appropriate corrections in the text**

 2) The di-nucleotide shuffling seems more adequate for RNA analysis.

**We now use the di-nucleotide shuffling to generate random RNA sequences.**

 3) In one paper1 the Triplexator software also was used for analysis of MEG3 RNA-DNA contacts. The comparison of the obtained results with the results of given manuscript should be provided. Seems in current manuscript a more accurate analysis with good controls was provided.

**In this original paper the authors focused on the triplex-based interactions of a single RNA (MEG3) with the chromatin. In the present study we are interested whether other transcripts may have a potential to interact with the same genomic regions. Taking into account the different aims (and approaches) of the studies we are not sure if it is reasonable to compare their results.**

 4) It would be good to look at the practice of using the program on literature and make sure that the program has a sufficiently low specificity.

**In our recent benchmarking study [ PMID:29697742] we showed that Triplexator was the most accurate tool as of 2018. This is why we used it in the present study.**

# Peer review: Reviewer response for version 1

**DOI:** 10.5256/f1000research.14683.r29954

**Published:** 2018-01-23

**Authors:** Ingrid Grummt

**Affiliations:** 1 Division of Molecular Biology of the Cell II , DKFZ-ZMBH-Allianz, German Cancer Research Center (DKFZ), Heidelberg, Germany

Long noncoding RNAs (lncRNA) can regulate gene expression by targeting specific DNA sequences via Hoogsteen base paring, forming RNA-DNA triple helical structures. Computational analyses have revealed that a large population of triplex-forming motifs is present across the genome, the majority of annotated human genes containing at least one unique and high-affinity triplex target site, preferentially in regulatory gene regions (Goni et al. 2004, Buske et al. 2012). Moreover, several studies have provided in vitro and in vivo evidence for the existence and biological relevance of RNA-DNA triplexes, including pRNA (Schmitz et al. 2010), Fendrr (Grote et al. 2013), Khps1 (Postepska-Igielska et al. 2015), PARTICLE (O’Leary et al. 2015), and MEG3 (Mondal et al. 2015). MEG3 has been shown to associate with AG-rich DNA motifs and facilitate recruitment of PRC2 to target sites. Considering the large number of purine-rich sequences in the genome, triplex-mediated targeting of lncRNAs and associated proteins to distinct genomic loci is very likely a commonly used mechanism of gene regulation.

 Given the importance and emerging acceptance of the concept of triplex-dependent gene regulation, it is more than surprising, if not irritating, that the authors challenge this concept feeding the ‘Triplexator’ only with a few RNAs and a subset of MEG3-interacting regions rather than providing any experimental data and/or more global bioinformatic analysis.

 Just some specific comments:

- In the abstract they claim ‘ *these triplex interactions might contribute to establishing long-distance chromosomal contact’* without providing any information or bioinformatic analyses.
- They use the term ‘hybridization’ for the interaction between RNA and dsDNA. This is wrong as hybridization refers to Watson-Crick base-pairing between RNA and ssDNA and not to Hoogsteen bonding.
- They took MEG3-interacting DNA peaks shorter than 1000 bp, then selected 3000 bp bins centering these regions and used these bins for analysis. There is no rationale for this selection which of course determines the final outcome of the analysis. Accordingly, the majority of these bin regions did not coincide with regions determined by ChOP-seq. Probably, a shorter binning would be more reliable to analyze the available data.

- They focused on bins that overlap genes. Even if partial overlapping was accepted, they might have missed some promoters. Intergenic regions containing regulatory sequences (e.g. enhancers) were excluded.
- Why were only peaks overlapping with annotated genes considered to be significant (or ”real“)? Genomic regions that do not harbor annotated genes, such as enhancers, are important regulatory elements that are targeted by lncRNAs and as such are functional RNA-binding sites, highly relevant for this study. In addition, since it is known that the base composition of genic and intergenic regions is different, exclusion of intergenic regions introduces a considerable bias to the analysis.
- Selection of just three additional RNAs is certainly not adequate for the far-reaching conclusion: ‘ *TTSs are able to hybridize with various different RNAs almost irrespectively of their sequence’*. It would be more convincing to show the results from scanning more RNAs, irrespective of their length and GC-content. Also, there is no attention given to the expression profiles of selected MEG3-mimicking RNAs. This is important because transcription of MEG3 is highly tissue-specific.
- The sum scores from the Triplexator analysis are shown which does not mean that the same regions are involved in triplex formation. It would be much more convincing to show similarities (or differences) of triplex-forming RNAs for a given TTS in a given bin.

- The terms ‘universal TTS’ and ‘universal bins’ are not synonymous and interchangeable!! One bin (3000 bp) can contain many putative TTSs.
- If there are only 18 ‘universal bins’ out of 3620 bins among seven RNA analyses, this small number is not sufficient for claiming that there is no specificity in RNA targeting.
- They hypothesize that *‘the universal TTS can be viewed as the anchor point which can be bound by various nuclear RNAs to provide long-distance chromosomal contacts’*. Even if this might be true, without any supportive data this is pure speculation.

 

 Altogether, the authors´ claim that triplex formation occurs almost sequence-independent is not justified but is based solely on *in silico* analyses. At least another available bioinformatics tool should have been used and standard *in vitro* assays (e.g. EMSA experiments) should have been performed to validate that the candidate RNAs are indeed capable to form triplexes. The authors do not even mention that the *in vivo* situation might be completely different than algorithm-based predictions and that there might be additional factors/constraints involved in triplex formation and stability.

I have read this submission. I believe that I have an appropriate level of expertise to state that I do not consider it to be of an acceptable scientific standard, for reasons outlined above.

# Response

**Published:** 2019-05-10

**Authors:** Ivan Antonov

**Affiliations:** Research Center of Biotechnology, RAS, Russian Federation

**We would like to thank Dr. Grummt for the extended comments to our work. They have helped us improve the design of our study and obtain additional results. We hope that they made our conclusions more reliable and reproducible.**

 Given the importance and emerging acceptance of the concept of triplex-dependent gene regulation, it is more than surprising, if not irritating, that the authors challenge this concept feeding the ‘Triplexator’ only with a few RNAs and a subset of MEG3-interacting regions rather than providing any experimental data and/or more global bioinformatic analysis.

**We do not challenge the possibility of triplex-dependant regulation. We simply claim that RNA interactions with some genomic regions have low sequence specificity because many other RNAs may be able to bind the same loci. We investigated features of such regions and found them to be enriched in purine rich low complexity repeats. In the current version, we completely redesigned the study and incorporated the analysis of 306 RNA sequences to confirm our findings. We also modified the text so the main conclusions are clear and non-misleading.**

 Just some specific comments:

 1) In the abstract they claim ‘these triplex interactions might contribute to establishing long-distance chromosomal contact’ without providing any information or bioinformatic analyses.

**We believe it is reasonable to speculate about this possibility in the discussion for the following reasons. First, it has recently been shown that "RNAs originating from super-enhancers form triplexes at distant regions" [PMID: 30605520]. Second, we showed that the predicted Capture-seq universal TTSs were highly enriched in gene promoters (Supplementary Figure 10). Together these observations indicate that the same eRNA may be able to interact with several different universal TTSs and therefore contribute to the long-distance chromosomal (i.e. enhancer-promoter) contacts. However, additional experimental verification of this hypothesis is required.**

**We modified the text in the abstract as follows: "We speculated that such universal TTSs may contribute to establishing long-distance chromosomal contacts and may facilitate distal enhancer-promoter interactions."**

 2) They use the term ‘hybridization’ for the interaction between RNA and dsDNA. This is wrong as hybridization refers to Watson-Crick base-pairing between RNA and ssDNA and not to Hoogsteen bonding.

**We have corrected the terminology used in the manuscript.**

 3) They took MEG3-interacting DNA peaks shorter than 1000 bp, then selected 3000 bp bins centering these regions and used these bins for analysis. There is no rationale for this selection which of course determines the final outcome of the analysis. Accordingly, the majority of these bin regions did not coincide with regions determined by ChOP-seq. Probably, a shorter binning would be more reliable to analyze the available data.

**We now use the exact locations for all the ChOP-seq peaks. To compensate for the peak length variability we developed a method that estimates the statistical significance of the number of triplexes predicted for a RNA-DNA pair taking into account lengths of both sequences.**

 4) They focused on bins that overlap genes. Even if partial overlapping was accepted, they might have missed some promoters. Intergenic regions containing regulatory sequences (e.g. enhancers) were excluded.

**We now analyze all the ChOP-seq peaks without considering their overlaps with the annotated genes.**

 5) Why were only peaks overlapping with annotated genes considered to be significant (or ”real“)? Genomic regions that do not harbor annotated genes, such as enhancers, are important regulatory elements that are targeted by lncRNAs and as such are functional RNA-binding sites, highly relevant for this study. In addition, since it is known that the base composition of genic and intergenic regions is different, exclusion of intergenic regions introduces a considerable bias to the analysis.

**We now analyze all the ChOP-seq peaks.**

 6) Selection of just three additional RNAs is certainly not adequate for the far-reaching conclusion: ‘TTSs are able to hybridize with various different RNAs almost irrespectively of their sequence’. It would be more convincing to show the results from scanning more RNAs, irrespective of their length and GC-content. Also, there is no attention given to the expression profiles of selected MEG3-mimicking RNAs. This is important because transcription of MEG3 is highly tissue-specific.

**We have increased the number of the considered query RNAs to 306 and used the expressed transcripts only.**

 7) The sum scores from the Triplexator analysis are shown which does not mean that the same regions are involved in triplex formation. It would be much more convincing to show similarities (or differences) of triplex-forming RNAs for a given TTS in a given bin.

 We no longer use sum scores as a measure of triplex-based interaction. Instead, we estimate the statistical significance (p-value) of each RNA-DNA interaction based on the number of predicted triplexes.

**Our work was focused on the properties of the DNA sequences that may allow them to interact with various different RNAs. We therefore analyzed the parts of the ChOP-seq/Capture-seq peaks universal TTSs that allowed them to interact with various different RNAs. Our analysis indicates that such triplex-forming hot-spots frequently coincide with the purine-rich low complexity genomic regions.**

 8) The terms ‘universal TTS’ and ‘universal bins’ are not synonymous and interchangeable!! One bin (3000 bp) can contain many putative TTSs.

**We do not use the concept of bins and ‘universal bins’ in the current version of the manuscript. However, we kept the term ‘universal TTS’.**

 9) If there are only 18 ‘universal bins’ out of 3620 bins among seven RNA analyses, this small number is not sufficient for claiming that there is no specificity in RNA targeting.

**We would like to emphasize that our paper don't question the concept of the sequence specific triplex-dependent gene regulation (moreover, we support this idea and conduct research in this direction). We claim that some genomic regions may have a potential to form triple helices with a variety of different long RNAs forming "universal" triplex target sites (TTSs). At the same time, we do not challenge the sequence specificificity of the other triplex-based RNA-DNA interactions.**

 10) They hypothesize that ‘the universal TTS can be viewed as the anchor point which can be bound by various nuclear RNAs to provide long-distance chromosomal contacts’. Even if this might be true, without any supportive data this is pure speculation.

 We agree that at the moment our claim is a hypothesis/speculation. Nevertheless, the recent published results [PMID: 30605520] as well our own indicate the possibility of such mechanism. By discussing it in the current manuscript we hope to attract attention of experimental biologists to further study this topic.

**We modified the text in the paper to clarify the issue:**

**"One of the possible and actively discussed roles of the chromatin bound RNAs (including lncRNAs) is to bring different chromosomal parts together to enable the remote DNA-DNA contacts. Moreover, it has recently been shown that RNAs originating from super-enhancers form triplexes at distant regions. Therefore, it is possible that universal TTSs may facilitate distal enhancer-promoter interactions via engagement with the same enhancer RNA. In line with this hypothesis, we observed the statistical significant enrichment of the universal Capture-**

**seq peaks near (< 1 kb) the transcription start sites (TSSs) of the annotated genes (Supplementary Figure 10C)."**

 11) Altogether, the authors´ claim that triplex formation occurs almost sequence-independent is not justified but is based solely on in silico analyses. At least another available bioinformatics tool should have been used and standard in vitro assays (e.g. EMSA experiments) should have been performed to validate that the candidate RNAs are indeed capable to form triplexes. The authors do not even mention that the in vivo situation might be completely different than algorithm-based predictions and that there might be additional factors/constraints involved in triplex formation and stability.

**We added analysis of the recent in vitro data obtained by the Capture-seq method. According to this experimental data some genomic fragments can interact with all three RNA oligos used in the original study. This supports the observations obtained in the analysis of the ChOP-seq peaks. Our computational analysis classified almost 40% of these shared Capture-seq peaks as universal TTSs. Moreover, the ChOP-seq and the Capture-seq universal TTSs were similar in that they were enriched with the purine rich low complexity regions. We believe that these results indirectly support the existence of universal TTS .  Yet, experimental validation of these results is beyond the scope of the current paper.**

**Still, at the end of the manuscript, we discuss the limitations of our computational approach and mention that the obtained results resemble mostly the situation with the naked DNA in vitro, than the interactions with the chromatin in vivo.**

**We added  the following text to the discussion:**

**"Importantly, the current computational analysis has a number of limitations. Namely, the triplex-based interactions of the full length transcripts were predicted without taking their secondary structure into account. We are not aware of any bioinformatics tools that would be able to produce such predictions. Moreover, cellular localization of the 153 selected expressed transcripts as well as DNA binding proteins and chromatin compaction were not considered. Therefore, our simulations are more similar to the in vitro Capture-seq experiments with short oligos than to the interactions of long transcripts with the chromatin inside the nucleus."**
